# Roosting and Foraging Social Structure of the Endangered Indiana Bat (*Myotis sodalis*)

**DOI:** 10.1371/journal.pone.0096937

**Published:** 2014-05-09

**Authors:** Alexander Silvis, Andrew B. Kniowski, Stanley D. Gehrt, W. Mark Ford

**Affiliations:** 1 Department of Fish and Wildlife Conservation, Virginia Polytechnic Institute and State University, Blacksburg, Virginia, United States of America; 2 School of Environment and Natural Resources, The Ohio State University, Columbus, Ohio, United States of America; 3 U.S. Geological Survey, Virginia Cooperative Fish and Wildlife Research Unit, Department of Fish and Wildlife Conservation, Virginia Polytechnic Institute and State University, Blacksburg, Virginia, United States of America; University of Regina, Canada

## Abstract

Social dynamics are an important but poorly understood aspect of bat ecology. Herein we use a combination of graph theoretic and spatial approaches to describe the roost and social network characteristics and foraging associations of an Indiana bat (*Myotis sodalis*) maternity colony in an agricultural landscape in Ohio, USA. We tracked 46 bats to 50 roosts (423 total relocations) and collected 2,306 foraging locations for 40 bats during the summers of 2009 and 2010. We found the colony roosting network was highly centralized in both years and that roost and social networks differed significantly from random networks. Roost and social network structure also differed substantially between years. Social network structure appeared to be unrelated to segregation of roosts between age classes. For bats whose individual foraging ranges were calculated, many shared foraging space with at least one other bat. Compared across all possible bat dyads, 47% and 43% of the dyads showed more than expected overlap of foraging areas in 2009 and 2010 respectively. Colony roosting area differed between years, but the roosting area centroid shifted only 332 m. In contrast, whole colony foraging area use was similar between years. Random roost removal simulations suggest that Indiana bat colonies may be robust to loss of a limited number of roosts but may respond differently from year to year. Our study emphasizes the utility of graphic theoretic and spatial approaches for examining the sociality and roosting behavior of bats. Detailed knowledge of the relationships between social and spatial aspects of bat ecology could greatly increase conservation effectiveness by allowing more structured approaches to roost and habitat retention for tree-roosting, socially-aggregating bat species.

## Introduction

Sociality is as an important contributor to day-roosting behavior in bats [1], [2] and roost fidelity is at least partly a group decision [3], [4]. The social systems for only a small number of bat species have been studied [1], [5], but individual bats within maternity colonies typically exhibit non-random social assortment dynamics [6]–[9]. Non–random social assortment among bats typically is reflected through coincident roost use [1] but association is not restricted solely to roosting behavior. Bats also are known to communicate roost and feeding site information [10], [11] and recognize familiar conspecifics during flight [12]. Although relationships between foraging and association have rarely been examined [13], these may also constitute an important part of bat sociality. Asynchronous roost use among individuals as a result of roost switching can, in some instances, result in social structures where individuals are both casual acquaintances and constant companions [8]. More generally, roost switching and asynchronous roost use results in a fission–fusion social system [1]. Fission-fusion social systems have been documented in several bat species [6], [7], [9], [14], [15]. These social systems are flexible and variable but often occur when the benefits of group membership are temporary [16]. Although it is unclear whether individuals form stronger associations with close relatives than less related individuals [17]–[19], maternity colonies of some bat species do appear to be based on matrilines [19]–[21]. There is also evidence indicating that reproductive condition impacts association and roost-mate choice at the individual level [15], [22]. At the colony level, social structure may be related to local roost availability and therefore dependent upon the spatio-temporal aspects of habitat quality and configuration [15], [23].

In North America, research on bat association largely has been limited to forested habitats and to relatively few species [7], [8], [14], [15], [19], [21], [22]. Notably, the social structure of the endangered Indiana bat (*Myotis sodalis*), whose Latin epithet *sodalis* (meaning companion) was selected due to an early perception of sociality [24], has not been described beyond classification as fission-fusion [25],[26]. Indiana bats are widely distributed across the eastern and midwestern United States [27] and form maternity colonies in summer wherein groups of bats generally roost beneath exfoliating bark of live trees or snags [26], [28]–[30]. Estimates vary but most maternity colonies appear to consist of fewer than 100 individuals [31] although >300 bats have been observed emerging from maternity roosts [32]. Over the summer maternity season female Indiana bats use multiple roost trees; however, usually 1 to 3 roosts receive consistent and/or repeated use by the majority of bats in the colony and are referred to as “primary roosts” [28]. The roosts used by individual or small numbers of bats intermittently or only for one to a few days are considered “secondary” or “alternate roosts.” Due to its perceived sociality, the Indiana bat potentially provides a model species to investigate the social dynamics of bats. Additionally, because of its endangered status and wide distribution across highly anthropogenically altered landscapes [27], [33], an improved understanding of Indiana bat social structure and roosting behavior could greatly benefit efforts to minimize impacts of human land use on the species and provide insight into habitat management efforts.

Observing associations and interactions between individual bats in species that roost under bark or in cavities and that switch roosts frequently, such as the Indiana bat, is extremely difficult. In previous studies of bat sociality [7], [8], [17], [34], association has been assessed using indices that document the amount of time that bats share roosts. An alternative approach however, is to infer association from use of a common resource [15], [35]. Network (graph theoretic) analysis offers a useful analytical framework, specifically two-mode networks, to assess such common resource use [36], [37]. As a special case of complex networks, two-mode networks contain two sets of nodes with connections only between nodes of the opposite set; in the study of bat sociality, bats and roosts can be considered separate sets of nodes [15], [35]. Analysis of the two-mode networks directly, or of one set of nodes individually through projection to a single-mode network, can be used to address questions regarding one or both sets of nodes [36]. Two-mode and other graph theoretic network analyses have been successfully used in animal studies to quantify social structure [38]–[40] and provide a broad framework for modeling and testing social, spatial and temporal hypotheses [40],[41].

In this study, we combined graph theoretic and spatial methods to: 1) describe the roosting social structure of the Indiana bat and 2) determine colony robustness to fragmentation as a result of roost loss through simulations. Secondarily, we assessed whether Indiana bats exhibit social foraging behavior and evaluated the overall size and stability of maternity colony roosting and foraging areas. Based on observed patterns of differential roost use by Indiana bats [28], [30], [42]–[44], we predicted that roost networks would be centralized whereas social networks would be decentralized. Because individuals of other tree-roosting bats form preferential associations [7], [8], [17], we further predicted that both roost and social networks would exhibit high clustering and modularity values indicative of the presence of preferentially associating cliques with preferred roosts [15], [45].

## Methods

### Study Site

**Figure 1:**
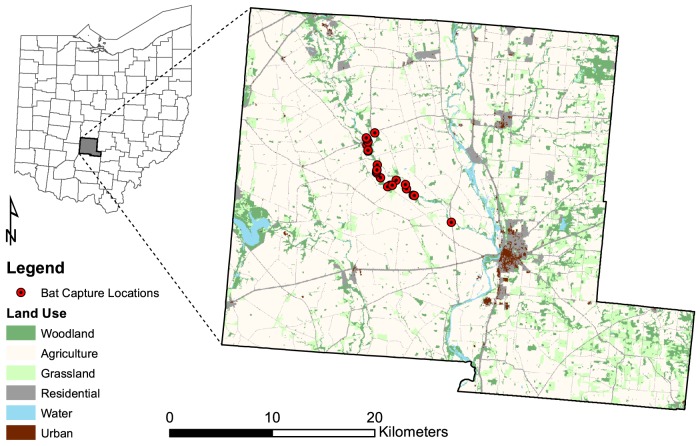
Indiana bat (*Myotis sodalis*) capture locations and landscape configuration in Pickaway County, Ohio, USA.

We conducted our study along Big Darby Creek in Pickaway County, Ohio, USA (Figure 1). Pickaway County is characterized by flat to gently rolling terrain with elevations ranging from 190 to 330 m above sea level. The Scioto River bisects the county and numerous smaller streams are present throughout. Cultivated cropland was the dominant land use within the county comprising 74% of the land area [46]. Woodlands composed 9% of the land area and were generally limited to field edges, creek banks, and small, widely scattered woodlots. Extant woodlots and/or riparian forests were commonly composed of box elder (*Acer negundo*), silver maple (*Acer saccharinum*), sugar maple (*Acer saccharum*), shagbark hickory (*Carya ovata*), common hackberry (*Celtis occidentalis*), white ash (*Fraxinus americana*) and American elm (*Ulmus americana*). We chose our general study location based on previously known Indiana bat roost locations with specific site locations determined by outreach to private landowners.

### Capture and Radio-tracking Methods

We captured Indiana bats using mist nets, 6–12 m in length and up to 8 m in height, at 21 sites along a 14–km section of Big Darby Creek. We placed mist nets across access roads, trails, along field edges, or across creek channels as conditions permitted. We attached radio transmitters (0.36 g, LB–2N, Holohil Systems Ltd., Carp, Ontario, Canada) between the scapulae of captured Indiana bats with surgical cement (Perma-Type, The Permatype Company Inc., Plainville, CT, USA) or eyelash adhesive (LashGrip, A.I.I., Los Angeles, CA, USA). All captured bats received lipped metal forearm bands (2.4 mm, Porzana Ltd., East Sussex, United Kingdom) and we recorded age, sex, and reproductive condition for each. We released bats at their capture site within 45 minutes of capture.

We recorded the diurnal roost location of each bat daily until the transmitter detached from the bat or the battery expired. We evaluated observation bias in the number of roosts used by individual bats by regressing the number of roosts used against the number of relocations. Because the number of roosts used is an integer count, we used a Poisson generalized linear model for our regression and evaluated the amount of deviance explained using maximum adjusted *D* ^2^ [47]. As permitted by logistical considerations (i.e., private property access and radio-tracking), we conducted emergence counts throughout the field season. We watched trees from dusk until five minutes after the last bat emerged or until insufficient light remained to see emerging bats. Due to logistical constraints, we were not always able to conduct exit counts on days when radiotagged bats were present but felt that such monitoring was appropriate given that we had no *a priori* reason to suspect that roosts could not be used when no radiotagged bats were present. We assessed whether patterns of roost use by radio-tagged bats were reflective of overall colony roost use by conducting correlation tests among the number of days a roost was used by radio-tagged bats and the maximum observed roost emergence.

When bats were active during the night, we estimated their locations in LOCATE III [48] using simultaneous bi- or tri-angulation bearings from mobile tracking stations. Three bearings per location were used most commonly—2 bearings/location comprised <3% of all locations. We used 3 or 4 element handheld yagi antennas and telemetry receivers (R2000, R4000, Advanced Telemetry Systems, Isanti, Minnesota, USA) to determine the most likely bearing from a given station to a bat. All azimuth bearings were recorded synchronously and 3 or more minutes apart for a given bat. We followed bats from the time they emerged in the evening until all bats in the tracking area roosted (generally 0000–0200 hours). We prioritized foraging location data collection each night based on transmitter age and amount of data previously collected so that foraging data was collected at a comparable level for each individual.

### Ethics statement

This study was carried out in accordance with state and federal requirements for capture and handling of endangered wildlife (Ohio Division of Wildlife wild animal permit number 11-297; United States Fish and Wildlife Service native endangered species recovery permit number TE06843A-0). Capture and handling protocol followed the guidelines of the American Society of Mammalogists [49] and was approved by the Ohio State University Institutional Animal Care and Use Committee (protocol number 2008A0102). Study sites were located on private lands which were accessed by explicit permission of the landowners. Data used in this study are archived in the Virginia Polytechnic Institute and State University VTechWorks institutional repository and are available at http://hdl.handle.net/10919/25802.

### Network Analysis

We defined an Indiana bat maternity colony as all female and juvenile bats connected by coincident roost use. We represented the colony graphically and analytically as a two-mode network that consisted of bats and roosts (hereafter roost network). We used this two-mode representation to assess patterns of roost use by the colony. We used the single-mode projection of the bat nodes (hereafter social network) to assess colony social structure as this provides a more generalized picture of how bats may associate than observations from the two-mode network. To reduce bias resulting from uneven tracking periods and observing only a portion of the colony, we did not assign edge weights. We assessed roost and social network structure using mean degree, network degree centralization, network density and clustering. We calculated degree centralization and density for roost and social networks using two-mode [36], [50] and single-mode formulations [45], [51], [52], respectively. For the social network only, we also used leading eigenvector modularity (hereafter modularity) [53], shortest path length [54], and tested for homophily [55] by age class. Degree centralization, density, and clustering have values between 0 and 1 (0 = low, 1 = high). These measures represent the extent that the network is structured around individual nodes and the distribution of connections between nodes [36], [45], [51], [52], [56]. Leading eigenvector modularity values range from 0 to 1 and provide a measure of how distinctly a network is separated into different communities [53], [57]. Average shortest path length is the average geodesic distance between any two nodes. Average shortest path length provides the social distance among individuals and therefore a measure of how information may flow through the network. Homophily values range from -1 to 1 with negative values indicating avoidance and positive values indicating preference for connections with individuals sharing a specific characteristic [55].

To determine whether our observed network values differed from those of random networks, we performed 500 Monte Carlo simulations and compared observed network metrics to random network metrics using two-tailed permutation tests [58], [59]. Because network metrics are dependent upon the size of an individual network, values from networks of differing size are challenging to compare [60]. Therefore, we generated our random networks with the same number of nodes as our observed networks and with a constant probability of link establishment using the Erdős-Réyni model [61], [62]. We used the *igraph* [63] and *tnet* libraries [50] in R [64] to visualize networks and calculate metrics. Our permutation tests were performed in R using a custom script with dependencies on the *igraph* and *tnet* libraries. We calculated all network values on a year-to-year basis because few individual bats were tracked during both years.

### Spatial Analysis

We assessed the potential for nightly association during flight by calculating the joint use of foraging space by dyads of bats using the utilization distribution overlap index (UDOI) [65]. The UDOI uses the joint distribution of two utilization distributions to assess spatial overlap relative to the volume of use within the area of overlap. UDOI values <1 indicate relatively uniform and independent space use, whereas values >1 indicate non-uniform space use with a high degree of overlap [65]. We generated foraging area utilization distributions for individual bats with ≥ 40 locations using biased random bridges (BRB) [66]. Use of BRBs allowed us to use serial autocorrelation in our foraging locations to better represent the actual movement pathways of bats. We estimated the diffusion parameter for BRB foraging utilization distributions using the plug-in method [66] with a maximum duration allowed between successive relocations of 60 minutes and a minimum distance between successive relocations of 50 meters. We used these same values in calculating the BRB where we set the minimum smoothing parameter to 88 meters and specified that the relocation variance have constant weight. We calculated the UDOI for all dyads of bats within years using the 95% BRB utilization distributions. We used a network map to visually represent the distribution of connections among bats given by overlapping foraging areas.

We also evaluated whole-colony space use using 95% utilization distributions calculated separately for roosting and foraging areas using the pooled locations of all bats. We calculated utilization distributions for the roosting and foraging areas using bivariate normal fixed kernel methodology. To reflect the concentration of roost use, we weighted roost locations by the number of days the roost was used by radio-tagged bats [34]. We used the reference method for smoothing parameter estimation as appropriate for weighted locations [67]. We assessed annual changes in roosting and foraging area use for the whole colony using Bhattacharya's affinity (BA) [65]. Similar to the UDOI, BA uses a joint distribution of two utilization distributions, but in contrast to the UDOI, quantifies similarity between utilization distributions and is more appropriate for comparisons of utilization distributions for the same individual or group [65]. BA values range from 0 to 1 with values close to 1 indicating highly similar utilization distributions [65]. To assess overall spatial drift in roosting area, we calculated the roosting area centroids in each year and the difference between these centroids. We calculated utilization distributions, BA and the UDOI using the *adehabitatHR* package [68] in R.

### Removal Simulation

We assessed the potential impact of roost loss on colony fragmentation using random and targeted node removal simulations [69]. We conducted these simulations using the single-mode projection of the roosting network to best reflect the knowledge that bats have of multiple roosts and their possible movement pathways between those roosts. We performed random removal simulations by iteratively removing an increasing number of random nodes until only 30% remained. Because the number of nodes differed between years, removal by percentage allowed us to compare the relative effects of roost loss between networks. We repeated removal simulations 1,000 times per proportion of nodes removed and calculated the mean and standard error of the number of resultant components; a component may be either a network fragment or an individual node. For our targeted removal simulation, we removed the most degree-central roost in the network and calculated the number of remaining components. Removal simulations were performed in R using a custom script with dependencies on the *igraph* and *tnet* libraries.

## Results

### Capture and Tracking

We captured 23 Indiana bats between 18 June and 30 August 2009, and 26 between 21 April and 6 August 2010. Of those bats captured in 2009, 14 were adult females (7 lactating, 2 post-lactating, 5 undetermined) and 7 were juveniles (3 male, 4 female). Twenty of the bats captured in 2010 were adult females (3 pregnant, 7 lactating, 2 post-lactating, 6 undetermined, 2 non-reproductive) and 5 were juveniles (2 male, 3 female). Mean transmitter mass (± SD) was 5.0 (± 0.8) percent of body mass in 2009 and 5.0 (± 0.7) percent in 2010. We recorded the roost location for 21 bats 195 times in 2009 and the roost location for 25 bats 228 times in 2010. These relocations represented 33 roosts in 2009 and 17 roosts in 2010; 7 roosts located in 2009 were also used in 2010. The mean (± SD) number of locations recorded per bat was 9.3 (± 5.0) in 2009 and 9.1 (± 5.4) in 2010. Roost switching occurred every 3.3 (± 1.4) days in 2009 and every 4.0 (± 3.1) days in 2010. The mean number of roosts used by a bat was 3.05 (± 1.77) in 2009 and 2.56 (± 1.33) in 2010. The number of roosts located per bat was weakly related to the number of locations recorded in both 2009 (β = 0.08, 95% CI: 0.0.03 – 0.13, *D^2^* = 0.49) and 2010 (β = 0.04, 95% CI: −0.00 – 0.09, *D^2^* = 0.19). The mean number of radiotagged bats that visited a roost was 1.94 (± 2.11) in 2009 and 3.76 (± 5.07) in 2010. We recorded 944 foraging locations for 16 bats in 2009 and 1362 foraging locations for 24 bats in 2010.

**Figure 2:**
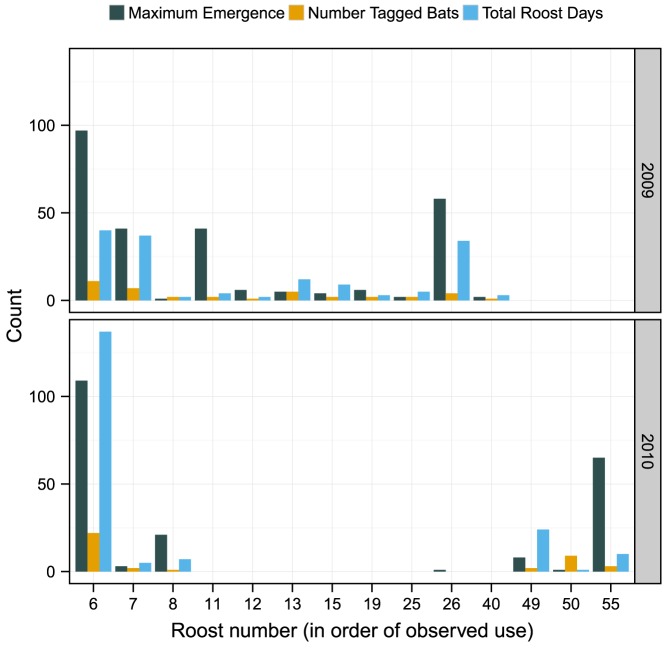
Indiana bat (*Myotis sodalis*) maternity roost uses. Maximum roost emergence, number of radio-tagged bats, and total roost days for roosts used by an Indiana bat maternity colony in Pickaway County, Ohio, USA, 2009–2010. Data are presented only for roosts with emergence counts.

We conducted exit counts at all 43 roosts; 11 and 7 roosts had non-zero counts in 2009 and 2010, respectively. Exit counts of zero occurred when exit counts were conducted on days when no radio-tagged bats were in the roost. Maximum emergence count was 97 in 2009 and 109 in 2010 (Figure 2). The maximum cumulative number of days an individual roost was used by radio-tagged bats was 40 in 2009 and 137 in 2010. The total number of days a roost was used by radio-tagged bats was positively correlated with the highest emergence count in both 2009 (r = 0.86, *P*<0.001) and 2010 (r = 0.85, *P*<0.001).

### Network Analysis

**Figure 3:**
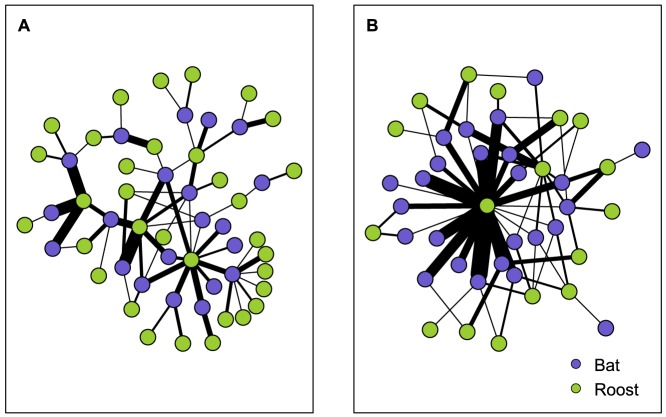
Indiana bat (*Myotis sodalis*) roost network maps. Two-mode network maps of an Indiana bat maternity colony in Pickaway County, Ohio, USA, 2009 (A) and 2010 (B). Node type indicated by color, edge width scaled by the number of connections.

Indiana bat roost network structure differed significantly from equivalent random networks and varied year-to-year (Table 1). Roost network density was low in 2009 indicating roosts were poorly connected overall (Figure 3). However, in 2010 roost network density was no different than random networks. The network was highly clustered in both years. That is, associate roosts (two nodes with a third node in common) were more frequently connected than in random networks suggesting roosts occurred in small but highly connected groups. Also, the network was more centralized than random networks in both years suggesting one or a small number of roosts were more connected and central within the network, although centralization was greater in 2010. Likewise, the observed mean number of uses of an individual roost was 5.91 (± 10.17) in 2009 and 13.41 (± 31.77) in 2010, indicative of a difference in network structure year-to-year.

**Table 1:** Indiana bat (*Myotis sodalis*) roost network metrics.

Year	Number of roosts	Mean degree	Density	Clustering	Degree centralization
2009	33	1.94	0.09 (<, 0.05)	0.61 (>, 0.002)	0.47 (>, 0.002)
2010	17	3.76	0.15 (0.42)	0.81(>, 0.002)	0.81 (>, 0.002)

Network metrics of an Indiana bat (*Myotis sodalis*) maternity colony roost network in Pickaway County, Ohio, USA, 2009–2010. Network metrics were calculated from a two-mode network consisting of bats and day-roosts. The direction of difference and *P*-values from Monte Carlo simulations are given in parenthesis.

**Figure 4:**
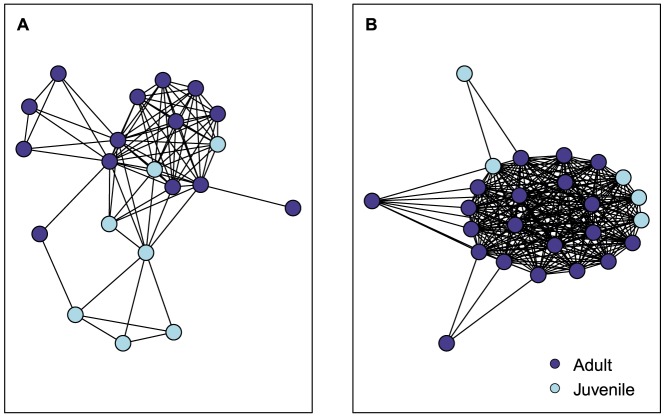
Indiana bat (*Myotis sodalis*) social network maps. Single-mode social network map of an Indiana bat maternity colony in Pickaway County, Ohio, USA, 2009 (A) and 2010 (B). Map projected from the two-mode network of bats and roosts. Nodes are colored by age class.

Indiana bat social network density and clustering were consistently greater than computed random social networks (Table 2) suggesting bats were more highly connected to one another than expected by chance (Figure 4). Network mean shortest path length was 1.8 in 2009 and 1.2 in 2010. We observed a high degree centralization value for the social network in 2009 indicating a small number of bats were better connected within the network. However, in 2010, degree centralization was no different than that of random networks suggesting that bats were equally connected throughout the network. Our modularity values indicate that the Indiana bat network contained no more modules than would be expected by chance occurrence in 2009 but fewer modules than would be expected by chance in 2010. Connections among bats were more homophilous than expected by age class in 2009, although the value was low. In contrast, homophily values were no different than those expected by chance in 2010.

**Table 2:** Indiana bat (*Myotis sodalis*) social network metrics.

Year	Number of bats	Mean degree	Density	Clustering	Degree centralization	Leading eigenvector modularity	Homophily
2009	21	8.00	0.40 (>, 0.002)	0.78 (>, 0.002)	0.44 (>, 0.006)	0.21 (0.54)	0.20 (>, 0.003)
2010	25	19.60	0.82 (>, 0.002)	0.96 (>, 0.002)	0.15 (0.14)	0.01 (<, 0.002)	−0.02 (0.10)

Network metrics of an Indiana bat (*Myotis sodalis*) maternity colony social network in Pickaway County, Ohio, USA, 2009–2010. Network metrics were calculated from single-mode projections of a two-mode network consisting of bats and day-roosts. The direction of difference and *P*-values from Monte Carlo simulations are given in parenthesis.

### Spatial Analysis

**Figure 5:**
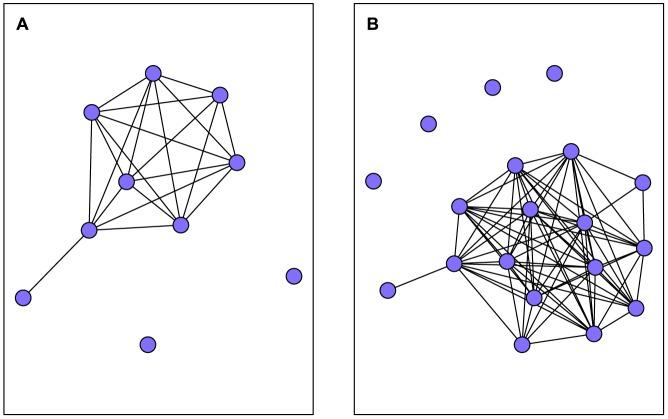
Indiana bat (*Myotis sodalis*) foraging network maps. Foraging network maps for of an Indiana bat maternity colony in Pickaway County, Ohio, USA in 2009 (A) and 2010 (B). Connections between nodes (bats) were created when the utilization distribution overlap index for a dyad was >1.

**Figure 6:**
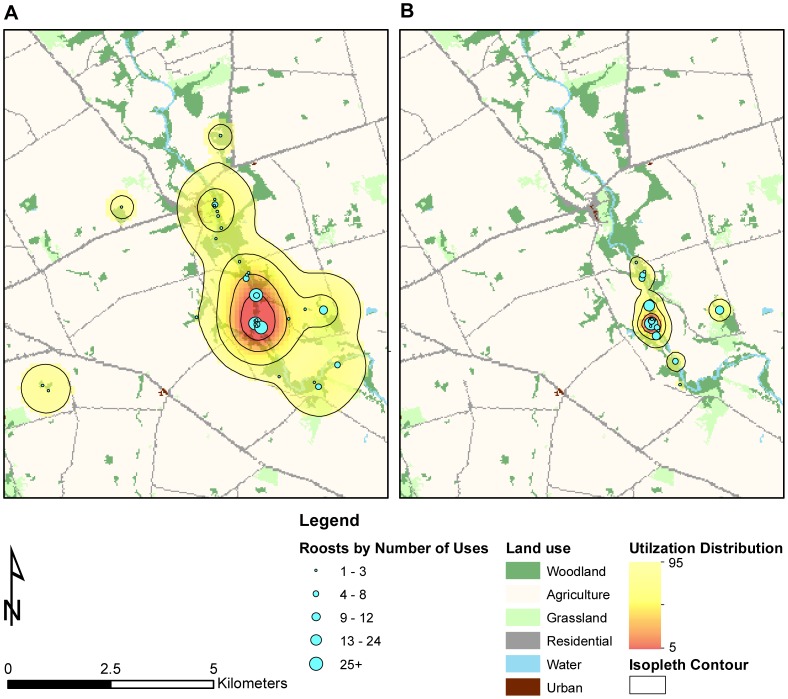
Indiana bat (*Myotis sodalis*) roosting areas. Bivariate fixed kernel density roosting area utilization distributions and day-roost locations of an Indiana bat (*Myotis sodalis*) maternity colony in Pickaway County, Ohio, USA in 2009 (A) and 2010 (B). Estimation of the utilization distributions was conducted using the pooled locations from all radio-tagged bats and weighted by the number of uses of individual roosts. Roost size is log scaled by the number of uses to show the relative contribution to the utilization distribution. The 25, 50, 75, and 95% home range contour intervals are shown.

We recorded ≥40 foraging telemetry locations for each of 10 bats in 2009 (representing 45 possible dyads) and 19 bats in 2010 (representing 171 possible dyads). We recorded an average of 70.9 (± 3.1) foraging locations per tracked individual. Overall mean BRB foraging range area was 376.0 ha (± 40.6) (individual home range and habitat selection of this colony was reported in Kniowski and Gehrt [33]). Twenty-one dyads in 2009 (47%) and 74 dyads in 2010 (43%) exhibited greater foraging area overlap than expected with the result that most bats shared foraging space with at least one other bat (Figure 5). Of those dyads with more than expected foraging area overlap, 11 (24% of total dyads) and 74 (43% of total dyads) also were in close proximity in the social network (i.e., shortest path lengths less than the colony average) in 2009 and 2010, respectively. Roost area for the entire colony was 1704.0 ha in 2009 and only 174.9 ha in 2010 (Figure 6), whereas foraging area was relatively constant at 3609.0 ha in 2009 and 3555.3 ha in 2010. Colony roosting area use differed between years (BA = 0.53); however, colony foraging area use did not differ as substantially (BA = 0.81). Despite the difference in overall colony roosting area between years, the roosting area centroids remained in approximately the same location (near the most central roost in the roost network) and differed only by 332 m.

### Roost Removal Simulation

**Figure 7:**
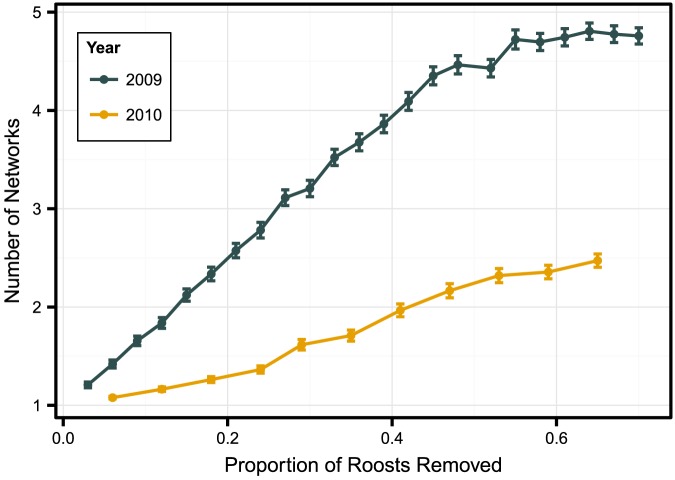
Roost removal impacts on Indiana bat (*Myotis sodalis*) network fragmentation. Simulated effect of roost removal on the fragmentation of an Indiana bat maternity colony roost network in Pickaway County, Ohio, USA, 2009–2010. Random roost removal was performed 1,000 times per proportion of roosts removed; lines represent mean ± standard error number of networks after node removal.

In 2009, the number of network fragments was linearly related to the proportion of roosts removed, with removal of approximately 5% of roosts generating a 50% chance (number of networks = 1.5) of network fragmentation (Figure 7). Similarly, the number of components was linearly related to the proportion of roosts removed in 2010, but less severely so as removal of approximately 30% of roosts was required to generate a 50% chance of network fragmentation. Targeted removal of the most central roost generated 4 network components in 2009 and 2 components in 2010.

## Discussion

Roost switching by Indiana bats resulted in formation of highly structured roosting and social networks. These networks differed significantly from random networks suggesting that the characteristics we observed were unlikely to have arisen by chance. As we predicted, roost networks were highly centralized relative to random networks whereas social networks were not. Social network modularity was low or no different than would be expected at random. Although the differences between our observed values and those of random networks were consistent across years, we found that some aspects of roost and social network structure and roosting space use differed between years. The most substantial differences include increased roost network centralization and connectedness among bats, a rarity of multiple-year use of roosts, and a concentration of roosting space use.

The differences in the network metrics between years for Indiana bats may be related to ecological factors such as roost quality, temperature [70], [71], suitability [26], [28]–[30], behavioral flexibility [15], or simply the result of tracking different individuals in each year. Because we tracked only three of the same bats in both years, our results do not necessarily indicate a change in network structure as differences may simply reflect different behavior by subsets of the colony at that site. However, the roosting behavior and social structure of bat maternity colonies may be inherently flexible and perhaps the differences between years such as we observed are common for the Indiana bat. Flexibility in roosting social behavior may be a useful adaptation given snag ephemerality [72]–[74] and has been documented in other species. For example, Silvis et al. [69] documented similar differences among the roosting and social networks of the northern bat (*Myotis septentrionalis*) and Johnson et al. [15] documented differences among network characteristics of Rafinesque's big-eared bat (*Corynorhinus rafinesquii*) in forested habitats.

In general, the high roost network degree centralization that we observed is consistent with the currently held idea that some Indiana bat roosts are “primary” and others “secondary.” In our study, the most central roost was the same in both 2009 and 2010. This roost was not only the most degree-central in the roost network, but it was used by the most radio-tagged bats, received the largest number of revisits, and had the largest exit counts. However, our emergence counts and other roost network structural characteristics indicate that the central roost did not necessarily contain the majority of bats within the colony at any given time. Distribution of the colony through multiple roosts, some of which at times contained substantial numbers of bats, suggests that exit counts of the primary roost provided limited information on colony size. Based on the distribution of bats throughout the roost network, even a large number of repeated counts at the primary roost will fail to account for bats that are spread consistently across multiple roosts. The small number of roosts located in 2009 that were re-used in 2010 also suggests that even if multiple roosts are monitored, exit counts may not convey the same ecological meaning across years. As such, understanding how bats are distributed through their roost network and how roosting behavior differs between years is necessary for robust estimation of colony size and evaluation of population trends from count data.

Little is known about secondary roost use by Indiana bats, but they appeared to be used selectively in our study. For bats generally, the use of multiple roosts is related to minimizing exposure to parasites [75], predator avoidance [76], and maintenance of social contacts [7]. For the Indiana bat, selective use of secondary roosts could help bats maintain preferred roosting companions, reflect incomplete individual knowledge of the roost network and greater communication within cliques, or possibly serve as a strategy for coping with roost loss. Because of our short tracking periods we were unable to fully document specific preferential associations among bats, but this has been shown in the congener northern bat [8] and may ensure a level of thermoregulatory stability. Despite selective use of secondary roosts, we found no evidence that Indiana bat maternity colony social networks had a modular structure. Indeed, our analysis indicates that bats within the colony largely were all in close social proximity as a result of coincident use of the most central roost in the roost network. Similarly, we found limited evidence that bats within age classes preferentially made connections with others in the same class. This suggests that our observed network structure was likely not a result of segregation of roosts by age class. Juvenile bats may have other impacts on aspects of network structure, and it is possible that network configuration changes with the addition of juvenile bats.

We found that nearly half of all Indiana bat dyads within years displayed more foraging overlap than expected, suggesting that association during foraging bouts may occur at some level. Echolocation calls such as “feeding buzzes” and other public foraging information are not believed to influence behavior of bats sufficiently to explain sociality [11], [77], but interaction during flight may function as a way to maintain social cohesion through recruitment of roost mates [2]. If association during foraging is important in recruitment of roost mates by Indiana bats, individuals or cliques within a maternity colony may be affected disparately by habitat disturbance in foraging areas. Similarly, if overlap of foraging areas is important in roost mate recruitment, disturbance of foraging areas may therefore also impact roosting network social structure and could pose a risk to persistence of maternity colonies. More foraging area overlap relative to the distribution of use within foraging ranges does not necessarily equate to association, and further may be an artifact of the location of the highest quality foraging habitat. However, a high level of overlap should be positively related to the potential for association.

The Indiana bat is known to exhibit strong fidelity to maternity roost areas [25], [78], [79]. The short distance between roosting area centroids and multi-year use of the most central roost in the roost network in our study support the idea of “general” roost area fidelity by Indiana bats in local landscapes. However, the number of roosts used in multiple years was limited and we did detect a substantial difference in the pattern of colony roost and space use between years. Why roost use and roost area differed so dramatically is unclear, but the more concentrated and spatially limited colony roosting area in 2010 is consistent with the increased roost network centralization in that year.

Given the ephemeral nature of roosts and the apparent relationship between roost network structure and roosting area, it seems likely that roosting areas could shift with roost loss (see also, [43]). Although little is known about how colony roost network structure and roosting area change in relation to roost deterioration and abandonment of primary roosts [80], we suggest the processes may be linked. For example, the colony may be scattered across numerous roosts in the season following abandonment of a primary roost as the colony locates and “chooses” another suitable primary roost. In following years, so long as the chosen primary roost remains suitable, the colony may concentrate roosting activity in the proximity of the new roost. Such a behavioral change is likely to be reflected in both network structure and roosting area use similar to what we observed. In central Indiana, Sparks et al. [81] found that Indiana bats used more roosts and congregated less the year following the natural loss of a single primary roost. Our data are limited temporally to only two years; however, there is evidence that the colony was using a different primary roost 2 years prior to our study (J. Chenger and K. Papenbrock, Bat Conservation and Management, Inc., unpublished report) and we may have observed part of the behavior change associated with the process of selecting a new primary roost. As the ephemerality of roost trees likely cause Indiana bat maternity colonies to experience frequent roost loss, including that of primary roosts, fission-fusion dynamics may provide a mechanism for the formation of new maternity colonies by presenting opportunities for the colony to split. Finally, habitat configuration may also influence changes in colony roosting area. Because our study was located in a highly agricultural area, forested habitats, and therefore likely also roosting resources, were limited and widely scattered across the landscape. In areas with greater amounts of forest or roosting resources, bats may not need to disperse as far in search of new roosts allowing a more stable roosting area. However, no information is currently available regarding whether roosting area is related to habitat configuration or resources.

Understanding roost area integrity and functionality is a primary concern in Indiana bat conservation. Our roost removal simulation results increase the understanding of how roost loss may impact bat colonies. Importantly, because it is possible to infer sociality from coincident roost use, fragmentation of the roost network also provides a method to begin to understand social aspects of colony fragmentation. In 2009 when we observed a less centralized roost network, connections between roosts, and by inference, between bats, in the colony were less robust to random roost loss than in 2010 when the network was more centralized. Similarly, the connections were more robust to targeted removal of the most central roost in 2010 than in 2009. The increased level of robustness to both random and targeted roost loss in 2010 was a result of a greater number of bats sharing secondary roosts. The greater number of connections allowed bats to maintain contact if a separate, shared roost was lost. In 2010, the level of colony social robustness to simulated random roost loss was greater than that of the northern bat [69] a species whose roosting ecology is frequently compared to that of the Indiana bat [26], [29], [82]. Conversely, in 2009 the Indiana bat colony was less robust to random roost loss than northern bat maternity colonies. Although our simulations suggest that in some instances Indiana bat colonies may fragment with the loss of a small proportion of roosts, this is based solely on the potential for association at the remaining roosts. Association by bats is not limited to roosts. Indeed, studies of bat communication [11] suggest it is possible for social connections to be re-established outside of roosts. In our study, foraging area overlap supports the idea that social connections could be re-established during foraging bouts. However, given the susceptibility to colony fragmentation that we observed in our simulations and the possible importance of foraging areas in the maintenance of colony structure, simultaneous loss of both roosting and foraging habitat likely has negative impacts on its social structure.

We did not incorporate roost specific (e.g., roost size, condition, or importance) and spatial factors (e.g. distribution of roosts) into our removal simulations, and therefore cannot predict certain network structural or spatial responses to specific roost loss. In particular, if individual roosts provide novel or highly preferred conditions for bats that are not replicated in another roost or potential roost, loss of that roost may cause changes in both association and space use. There is evidence that individual roosts are important and cannot be easily replaced in some bat species [83], [84], but the severity of impact caused by roost loss may be related to the degree of resource specialty and availability [84]. We suggest that Indiana bat colonies could respond to loss of an irreplaceable roost in several ways: 1) the entire colony could relocate to a nearby area with suitable replacement roosts; 2) the colony may fragment and occupy multiple areas or merge with other colonies reducing requirements for local roosting resources; or 3) the roosting area used by the colony could increase in size to incorporate suitable replacement roosts. Indiana bat colonies in the Midwest exhibit strong site fidelity [25], [78], [79] and it is not known to what extent Indiana bats are able to relocate geographically even if suitable replacement roosts exist in another area. Site fidelity appears to be more variable in the Appalachians however [30], and it is possible that colonies readily are able to relocate when roosting and foraging habitats are abundant. How or if multiple Indiana bat maternity colonies are able to coexist or merge is unknown, but this dynamic also may vary with the level of roost availability in a region. In the case of the loss of an irreplaceable roost where no adequate replacement exists and the colony does not relocate, there is risk that the colony becomes a non-contributing sink due to the impact of inadequate resources.

## Conclusions

To our knowledge, we are the first to describe the roosting social structure and foraging associations of the Indiana bat and our study highlights the utility of examining sociality and roosting behavior of bats through a combination of graph theoretic and spatial methods. Our results support currently held ideas that Indiana bat maternity colonies utilize roosts differentially and are loyal to roosting areas but also highlight a level of complexity in both roost and roosting area use that has not been previously described. Further, our study raises questions about the resiliency of Indiana bats to roost loss at ‘primary’ and ‘secondary’ roosts. Identifying how Indiana bat maternity colonies incorporate structured, non-random use of ephemeral roosting resources with relatively stable foraging areas remains a critical component for conservation planning. In addition, understanding colony mobility within landscapes in response to roost availability is critical for conservation and management of this endangered species as well as other bat species. Although our study begins to address the issues of how Indiana bat maternity colonies are structured socially and spatially, our understanding of the interactions between roost network structure, habitat, and geographical space use remains greatly limited. Additionally, because we did not track adult males, our work addresses only part of the Indiana bat population. The social structure of adult males remains an enigma, but it seems unlikely that our results will apply. Replication of this study with longer duration and greater sample size across different habitat types is needed to fully describe these processes. Identifying the similarities and differences in colony structure across an array of geographic locations and habitat configurations would provide insight into the biological and ecological factors influencing colony behavior. For example, in contrast to our study, Indiana bat maternity colonies located in roost abundant areas may be less closely associated and more mobile on the landscape as suitable roosts are widely distributed spatially. Finally, manipulative experiments involving roost removal in conjunction with roost removal simulations could add greatly to the utility of the simulations and our understanding of colony structure and ecology. Although such experiments may have been possible in conjunction with habitat alteration work in previous years, reductions in population size due to white-nose syndrome [85], [86] probably preclude such opportunities for the foreseeable future. Experimental manipulations on common species with similar roosting requirements and social behavior may provide useful information for conservation of the Indiana bat and other rare bat species.

## References

[1] Kerth G (2008) Causes and consequences of sociality in bats. BioScience 58: 737–746 10.1641/B580810

[2] Chaverri G, Gillam EH, Kunz TH (2013) A call-and-response system facilitates group cohesion among disc-winged bats. Behav Ecol 24: 481–487 10.1093/beheco/ars188

[3] Kerth G, Reckardt K (2003) Information transfer about roosts in female Bechstein's bats: an experimental field study. Proc R Soc Lond B Biol Sci 270: 511–515 10.1098/rspb.2002.2267 PMC1691266 12641906

[4] Kerth G, Ebert C, Schmidtke C (2006) Group decision making in fission–fusion societies: evidence from two-field experiments in Bechstein's bats. Proc R Soc B Biol Sci 273: 2785–2790 10.1098/rspb.2006.3647 PMC1635504 17015328

[5] Johnson JS, Kropczynski JN, Lacki MJ (2013) Social network analysis and the study of sociality in bats. Acta Chiropterologica 15: 1–17 10.3161/150811013X667821

[6] Kerth G, Konig B (1999) Fission, fusion and nonramdon associations in female Bechstein's bats (*Myotis bechsteinii*). Behaviour 136: 1187–1202 10.1163/156853999501711

[7] Willis CKR, Brigham RM (2004) Roost switching, roost sharing and social cohesion: forest-dwelling big brown bats, *Eptesicus fuscus*, conform to the fission–fusion model. Anim Behav 68: 495–505 10.1016/j.anbehav.2003.08.028

[8] Garroway CJ, Broders HG (2007) Nonrandom association patterns at northern long-eared bat maternity roosts. Can J Zool 85: 956–964 10.1139/Z07-079

[9] Rhodes M (2007) Roost fidelity and fission-fusion dynamics of white-striped free-tailed bats (*Tadarida australis*). J Mammal 88: 1252–1260.

[10] Wilkinson GS (1992) Information transfer at evening bat colonies. Anim Behav 44: 501–518 10.1016/0003-3472(92)90059-I

[11] Jonker MN, Boer WFD, Kurvers RHJM, Dekker JJA (2010) Foraging and public information use in common pipistrelle bats (*Pipistrellus pipistrellus*): a field experiment. Acta Chiropterologica 12: 197–203 10.3161/150811010X504699

[12] Mann O, Lieberman V, Köhler A, Korine C, Hedworth HE, et al (2011) Finding your friends at densely populated roosting places: male Egyptian fruit bats (*Rousettus aegyptiacus*) distinguish between familiar and unfamiliar conspecifics. Acta Chiropterologica 13: 411–417 10.3161/150811011X624893

[13] Chaverri G, Gamba-Rios M, Kunz TH (2007) Range overlap and association patterns in the tent-making bat *Artibeus watsoni* . Anim Behav 73: 157–164 10.1016/j.anbehav.2006.06.003

[14] Johnson JB, Ford WM, Edwards JW (2012) Roost networks of northern myotis (*Myotis septentrionalis*) in a managed landscape. For Ecol Manag 266: 223–231 10.1016/j.foreco.2011.11.032

[15] Johnson JS, Kropczynski JN, Lacki MJ, Langlois GD (2012) Social networks of Rafinesque's big-eared bats (*Corynorhinus rafinesquii*) in bottomland hardwood forests. J Mammal 93: 1545–1558 10.1644/12-MAMM-A-097.1

[16] Aureli F, Schaffner CM, Boesch C, Bearder SK, Call J, et al (2008) Fission-fusion dynamics: New research frameworks. Curr Anthropol 49: 627–654.

[17] Kerth G, Perony N, Schweitzer F (2011) Bats are able to maintain long-term social relationships despite the high fission–fusion dynamics of their groups. Proc R Soc B Biol Sci 278: 2761–2767 10.1098/rspb.2010.2718 PMC3145188 21307051

[18] Kozhurina EI (1993) Social organization of a maternity group in the noctule bat, *Nyctalus* noctula (Chiroptera: Vespertilionidae). Ethology 93: 89–104 10.1111/j.1439-0310.1993.tb00981.x

[19] Patriquin KJ, Palstra F, Leonard ML, Broders HG (2013) Female northern myotis (*Myotis septentrionalis*) that roost together are related. Behav Ecol 24: 949–954 10.1093/beheco/art012

[20] Kerth G (2008) Animal sociality: bat colonies are founded by relatives. Curr Biol 18: R740–R742 10.1016/j.cub.2008.07.038 18786373

[21] Metheny J, Kalcounis-Rueppell M, Willis C, Kolar K, Brigham R (2008) Genetic relationships between roost-mates in a fission–fusion society of tree-roosting big brown bats (*Eptesicus fuscus*). Behav Ecol Sociobiol 62: 1043–1051 10.1007/s00265-007-0531-y

[22] Patriquin KJ, Leonard ML, Broders HG, Garroway CJ (2010) Do social networks of female northern long-eared bats vary with reproductive period and age? Behav Ecol Sociobiol 64: 899–913 10.1007/s00265-010-0905-4

[23] Chaverri G, Kunz TH (2010) Ecological determinants of social systems: perspectives on the functional role of roosting ecology in the social behavior of tent-roosting bats. Behavioral ecology of tropical animals. Academic Press, Vol. Volume 42. pp. 275–318.

[24] Miller GS Jr, Allen GM (1928) The American bats of the genera *Myotis* and *Pizonyx* . Bull US Natl Mus 144: 1–218.

[25] Gumbert MW, O’keefe JM, MacGregor JR (2002) Roost fidelity in Kentucky. The Indiana bat: biology and management of an endangered species. Austin, Texas, USA: Bat Conservation International. pp. 143–152.

[26] Carter TC, Feldhamer GA (2005) Roost tree use by maternity colonies of Indiana bats and northern long-eared bats in southern Illinois. For Ecol Manag 219: 259–268 10.1016/j.foreco.2005.08.049

[27] Gardner JE, Cook EA (2002) Seasonal and geographic distribution and quantification of potential summer habitat. In: Kurta A, Kennedy J, editors. The Indiana bat: biology and management of an endangered species. Austin, Texas, USA: Bat Conservation International. pp. 9–20.

[28] Callahan EV, Drobney RD, Clawson RL (1997) Selection of Summer Roosting Sites by Indiana Bats (*Myotis sodalis*) in Missouri. J Mammal 78: 818–825 10.2307/1382939

[29] Foster RW, Kurta A (1999) Roosting ecology of the northern bat (*Myotis septentrionalis*) and comparisons with the endangered Indiana bat (*Myotis sodalis*). J Mammal 80: 659–672 10.2307/1383310

[30] Britzke ER, Harvey MJ, Loeb SC (2003) Indiana bat, *Myotis sodalis*, maternity roosts in the southern United States. Southeast Nat 2: 235–242 doi:10.1656/1528-7092(2003)002[0235:IBMSMR]2.0.CO;2

[31] Harvey MJ (2002) Status and ecology in the southern United States. In: Kurta A, Kennedy J, editors. The Indiana bat: biology and management of an endangered species. Austin, Texas, USA: Bat Conservation International. pp. 29–34.

[32] Whitaker JO, Brack V (2002) Distribution and summer ecology in Indiana. In: Kurta A, Kennedy J, editors. The Indiana bat: biology and management of an endangered species. Austin, Texas, USA: Bat Conservation International. pp. 48–54.

[33] Kniowski AB, Gehrt SD (2014) Home range and habitat selection of the Indiana bat in an agricultural landscape. J Wildl Manag 78: 503–512 10.1002/jwmg.677

[34] Popa-Lisseanu AG, Bontadina F, Mora O, Ibáñez C (2008) Highly structured fission–fusion societies in an aerial-hawking, carnivorous bat. Anim Behav 75: 471–482 10.1016/j.anbehav.2007.05.011

[35] Fortuna MA, Popa-Lisseanu AG, Ibáñez C, Bascompte J (2009) The roosting spatial network of a bird-predator bat. Ecology 90: 934–944 10.2307/25592582 19449689

[36] Borgatti SP, Everett MG (1997) Network analysis of 2-mode data. Soc Netw 19: 243–269 10.1016/S0378-8733(96)00301-2

[37] Williams RJ (2011) Biology, methodology or chance? The degree distributions of bipartite ecological networks. PLoS ONE 6: e17645 10.1371/journal.pone.0017645 21390231 PMC3048397

[38] Lusseau D, Newman MEJ (2004) Identifying the role that animals play in their social networks. Proc R Soc B Biol Sci 271: S477–S481 10.1098/rsbl.2004.0225 PMC1810112 15801609

[39] Sih A, Hanser SF, McHugh KA (2009) Social network theory: new insights and issues for behavioral ecologists. Behav Ecol Sociobiol 63: 975–988 10.1007/s00265-009-0725-6

[40] Wey T, Blumstein DT, Shen W, Jordán F (2008) Social network analysis of animal behaviour: a promising tool for the study of sociality. Anim Behav 75: 333–344 10.1016/j.anbehav.2007.06.020

[41] Jacoby DMP, Brooks EJ, Croft DP, Sims DW (2012) Developing a deeper understanding of animal movements and spatial dynamics through novel application of network analyses. Methods Ecol Evol 3: 574–583 10.1111/j.2041-210X.2012.00187.x

[42] Kurta A, King D, Teramino J, Stribley J, Williams K (1993) Summer roosts of the endangered Indiana bat (*Myotis sodalis*) on the northern edge of its range. Am Midl Nat 129: 132–138.

[43] Kurta A, Murray SW, Miller DH (2002) Roost selection and movements across the summer landscape. In: Kurta A, Kennedy J, editors. Bat Conservation International. pp. 118–129.

[44] Miller NE, Drobney RD, Clawson RL, Callahan EV (2002) Summer habitat in northern Missouri. In: Kurta A, Kennedy J, editors. Bat Conservation International. pp. 165–171.

[45] Watts DJ, Strogatz SH (1998) Collective dynamics of “small-world” networks. Nature 393: 440–442 10.1038/30918 9623998

[46] Homer C, Huang C, Yang L, Wylie B, Coan M (2004) Development of a 2001 national landcover database for the United States. Photogramm Eng Remote Sens 70: 829–840.

[47] Guisan A, Zimmermann NE (2000) Predictive habitat distribution models in ecology. Ecol Model 135: 147–186 10.1016/S0304-3800(00)00354-9

[48] Nams VO (2006) Locate III User's Guide. Tatamagouche, Nova Scotia, Canada: Pacer Computer Software.

[49] Sikes RS, Gannon WL (2011) the Animal Care and Use Committee of the American Society of Mammalogists (2011) Guidelines of the American Society of Mammalogists for the use of wild mammals in research. J Mammal 92: 235. 10.1093/jmammal/gyw078 PMC5909806 29692469

[50] Opsahl T (2009) Structure and evolution of weighted networks [Dissertation]. London, United Kingdom: Queen Mary, University of London.

[51] Freeman LC (1978) Centrality in social networks conceptual clarification. Soc Netw 1: 215–239 10.1016/0378-8733(78)90021-7

[52] Wasserman S, Faust K (1994) Social network analysis: methods and applications. Cambridge University Press. 852 p.

[53] Newman MEJ (2006) Finding community structure in networks using the eigenvectors of matrices. Phys Rev E 74: 036104 10.1103/PhysRevE.74.036104 17025705

[54] West DB (1996) Introduction to graph theory. Upper Saddle River, NJ: Prentice Hall Inc.

[55] McPherson M, Smith-Lovin L, Cook JM (2001) Birds of a feather: homophily in social networks. Annu Rev Sociol 27: 415–444 10.2307/2678628

[56] Dong J, Horvath S (2007) Understanding network concepts in modules. BMC Syst Biol 1: 24 10.1186/1752-0509-1-24 17547772 PMC3238286

[57] Newman MEJ (2006) Modularity and community structure in networks. Proc Natl Acad Sci 103: 8577–8582 10.1073/pnas.0601602103 16723398 PMC1482622

[58] Hope ACA (1968) A simplified Monte Carlo significance test procedure. J R Stat Soc Ser B Methodol 30: 582–598 10.2307/2984263

[59] Davison AC (1997) Bootstrap methods and their application. Cambridge, New York: Cambridge University Press. 598 p.

[60] James R, Croft DP, Krause J (2009) Potential banana skins in animal social network analysis. Behav Ecol Sociobiol 63: 989–997 10.1007/s00265-009-0742-5

[61] Erdős P, Rényi A (1960) On the evolution of random graphs. Math Inst Hung Acad Sci 5: 17–61.

[62] Newman M (2006) The structure and dynamics of networks: Princeton, New Jersey: Princeton University Press. 596 p.

[63] Csardi G, Nepusz T (2006) The igraph software package for complex network research. InterJournal Complex Systems: 1695.

[64] R. Development Core Team (2011) R: A language and environment for statistical computing. Vienna, Austria. Available: http://www.R-project.org/.

[65] Fieberg J, Kochanny CO (2005) Quantifying home-range overlap: the importance of the utilization distribution. J Wildl Manag 69: 1346–1359 doi:10.2193/0022-541X(2005)69[1346:QHOTIO]2.0.CO;2

[66] Benhamou S (2011) Dynamic approach to space and habitat use based on biased random bridges. PLoS ONE 6: e14592 10.1371/journal.pone.0014592 21297869 PMC3027622

[67] Gitzen RA, Millspaugh JJ, Kernohan BJ (2006) Bandwidth selection for fixed-kernel analysis of animal utilization distributions. J Wildl Manag 70: 1334–1344 doi:;10.2193/0022-541X(2006)70[1334:BSFFAO]2.0.CO;2

[68] Calenge C (2006) The package “adehabitat” for the R software: A tool for the analysis of space and habitat use by animals. Ecol Model 197: 516–519 10.1016/j.ecolmodel.2006.03.017

[69] Silvis A, Ford WM, Britzke ER, Johnson JB (2014) Association, roost use and simulated disruption of *Myotis septentrionalis* maternity colonies. Behav Processes 103: 283–290 10.1016/j.beproc.2014.01.016 24468215

[70] Humphrey SR, Richter AR, Cope JB (1977) Summer Habitat and Ecology of the Endangered Indiana Bat, *Myotis sodalis* . J Mammal 58: 334–346 10.2307/1379332

[71] Willis C, Brigham R (2007) Social thermoregulation exerts more influence than microclimate on forest roost preferences by a cavity-dwelling bat. Behav Ecol Sociobiol 62: 97–108 10.1007/s00265-007-0442-y

[72] Moorman CE, Russell KR, Sabin GR, Guynn Jr DC (1999) Snag dynamics and cavity occurrence in the South Carolina Piedmont. For Ecol Manag 118: 37–48 10.1016/S0378-1127(98)00482-4

[73] Bagne KE, Purcell KL, Rotenberry JT (2008) Prescribed fire, snag population dynamics, and avian nest site selection. For Ecol Manag 255: 99–105 10.1016/j.foreco.2007.08.024

[74] Wisdom MJ, Bate LJ (2008) Snag density varies with intensity of timber harvest and human access. For Ecol Manag 255: 2085–2093 10.1016/j.foreco.2007.12.027

[75] Reckardt K, Kerth G (2007) Roost selection and roost switching of female Bechstein's bats (*Myotis bechsteinii*) as a strategy of parasite avoidance. Oecologia 154: 581–588 10.1007/s00442-007-0843-7 17805579

[76] Kunz TH, Lumsden LF (2003) Ecology of cavity and foliage roosting bats. In: Kunz TH, Fenton MB, editors. Bat Ecology. Chicago, Illinois: University of Chicago Press. pp. 2–90.

[77] Kerth G, Wagner M, König B (2001) Roosting together, foraging apart: information transfer about food is unlikely to explain sociality in female Bechstein's bats (*Myotis bechsteinii*). Behav Ecol Sociobiol 50: 283–291 10.1007/s002650100352

[78] Kurta A, Murray SW (2002) Philopatry And migration of banded Indiana bats (*Myotis sodalis*) and effects Of radio transmitters. J Mammal 83: 585–589.

[79] Sparks DW, Whitaker JO Jr, Ritzi CM (2005) Foraging ecology of the endangered Indiana bat. In: Vories KC, Harrington A, editors. Office of Surface Mining, U.S. Department of the Interior. pp. 15–27.

[80] Kurta A (2005) Roosting ecology and behavior of Indiana bats (*Myotis sodalis*) in summer. In: Vories KC, Harrington A, editors. The Indiana bat and coal mining. Alton, Illinois, USA: Office of Surface Mining, U. S. Department of the Interior. pp. 29–42.

[81] Sparks DW, Simmons MT, Gummer CL, Duchamp JE (2003) Disturbance of roosting bats by woodpeckers and raccoons. Northeast Nat 10: 105–108 doi:10.1656/1092-6194(2003)010[0105:DORBBW]2.0.CO;2

[82] Timpone JC, Boyles JG, Murray KL, Aubrey DP, Robbins LW (2009) Overlap in Roosting Habits of Indiana Bats (*Myotis sodalis*) and Northern Bats (*Myotis septentrionalis*). Am Midl Nat 163: 115–123 10.1674/0003-0031-163.1.115

[83] Brigham RM, Fenton MB (1986) The influence of roost closure on the roosting and foraging behaviour of *Eptesicus fuscus* (Chiroptera: Vespertilionidae). Can J Zool 64: 1128–1133 10.1139/z86-169

[84] Chaverri G, Kunz TH (2011) Response of a specialist bat to the loss of a critical resource. PLoS ONE 6: e28821 10.1371/journal.pone.0028821 22216118 PMC3244425

[85] Minnis AM, Lindner DL (2013) Phylogenetic evaluation of *Geomyces* and allies reveals no close relatives of *Pseudogymnoascus destructans*, comb. nov., in bat hibernacula of eastern North America. Fungal Biol 117: 638–649 10.1016/j.funbio.2013.07.001 24012303

[86] Blehert DS, Hicks AC, Behr M, Meteyer CU, Berlowski-Zier BM, et al (2009) Bat White-Nose Syndrome: An Emerging Fungal Pathogen? Science 323: 227–227 10.1126/science.1163874 18974316

